# estiMAge: development of a DNA methylation clock to estimate the methylation age of single cells

**DOI:** 10.1093/bioadv/vbaf005

**Published:** 2025-01-16

**Authors:** Zoe Saßmannshausen, Lisa Blank, Llorenç Solé-Boldo, Frank Lyko, Günter Raddatz

**Affiliations:** Division of Epigenetics, DKFZ-ZMBH Alliance, German Cancer Research Center, D-69120 Heidelberg, Germany; Division of Epigenetics, DKFZ-ZMBH Alliance, German Cancer Research Center, D-69120 Heidelberg, Germany; Division of Epigenetics, DKFZ-ZMBH Alliance, German Cancer Research Center, D-69120 Heidelberg, Germany; Division of Epigenetics, DKFZ-ZMBH Alliance, German Cancer Research Center, D-69120 Heidelberg, Germany; Division of Epigenetics, DKFZ-ZMBH Alliance, German Cancer Research Center, D-69120 Heidelberg, Germany

## Abstract

**Motivation:**

Since their introduction about 10 years ago, methylation clocks have provided broad insights into the biological age of different species, tissues, and in the context of several diseases or aging. However, their application to single-cell methylation data remains a major challenge, because of the inherent sparsity of such data, as many CpG sites are not covered. A methylation clock applicable on single-cell level could help to further disentangle the processes that drive the ticking of epigenetic clocks.

**Results:**

We have developed estiMAge (“estimation of Methylation Age”), a framework that exploits redundancy in methylation data to substitute missing CpGs of trained methylation clocks in single cells. Using Euclidean distance as a measure of similarity, we determine which CpGs covary with the required CpG sites of an epigenetic clock and can be used as surrogates for clock CpGs not covered in single-cell experiments. estiMAge is thus a tool that can be applied to standard epigenetic clocks built on elastic net regression, to achieve bulk and single-cell resolution. We show that estiMAge can accurately predict the ages of young and old hepatocytes and can be used to generate single-cell versions of publicly available epigenetic clocks.

**Availability and implementation:**

The source code and instructions for usage of estiMAge are available at https://github.com/DivEpigenetics/estiMAge

## 1 Introduction

Since their introduction 10 years ago, DNA methylation clocks have become an integral part of the analysis of epigenetic data. While the primary purpose of these clocks in their early days was to predict the chronological age of a sample, in recent years, other applications have been explored, most notably estimating a subject’s biological age or the likelihood of developing certain diseases (Horvath and Raj 2018, Levine *et al.* 2018, Lu *et al.* 2019). Having become an increasingly used biomarker of aging methylation clocks have developed in numerous new directions of research. Important examples include studies about the modular structure of methylation clocks (Levine et al. 2022, Haghani *et al.* 2023), the development of species universal clocks (Lu *et al.* 2023), the analysis of clocks by simulations (Okada 2024, Tarkhov *et al.* 2024), or by advanced biostatistical methods to answer the question to what extent clock CpGs reflect causality or pure correlation (Ying *et al.* 2024a). Besides, new technologies for efficient quantification of clocks have been developed (Griffin *et al.* 2024) accompanied by suggestions to standardize the performance measures of clocks applied as biomarkers (Moqri *et al.* 2023).

**Figure 1.**
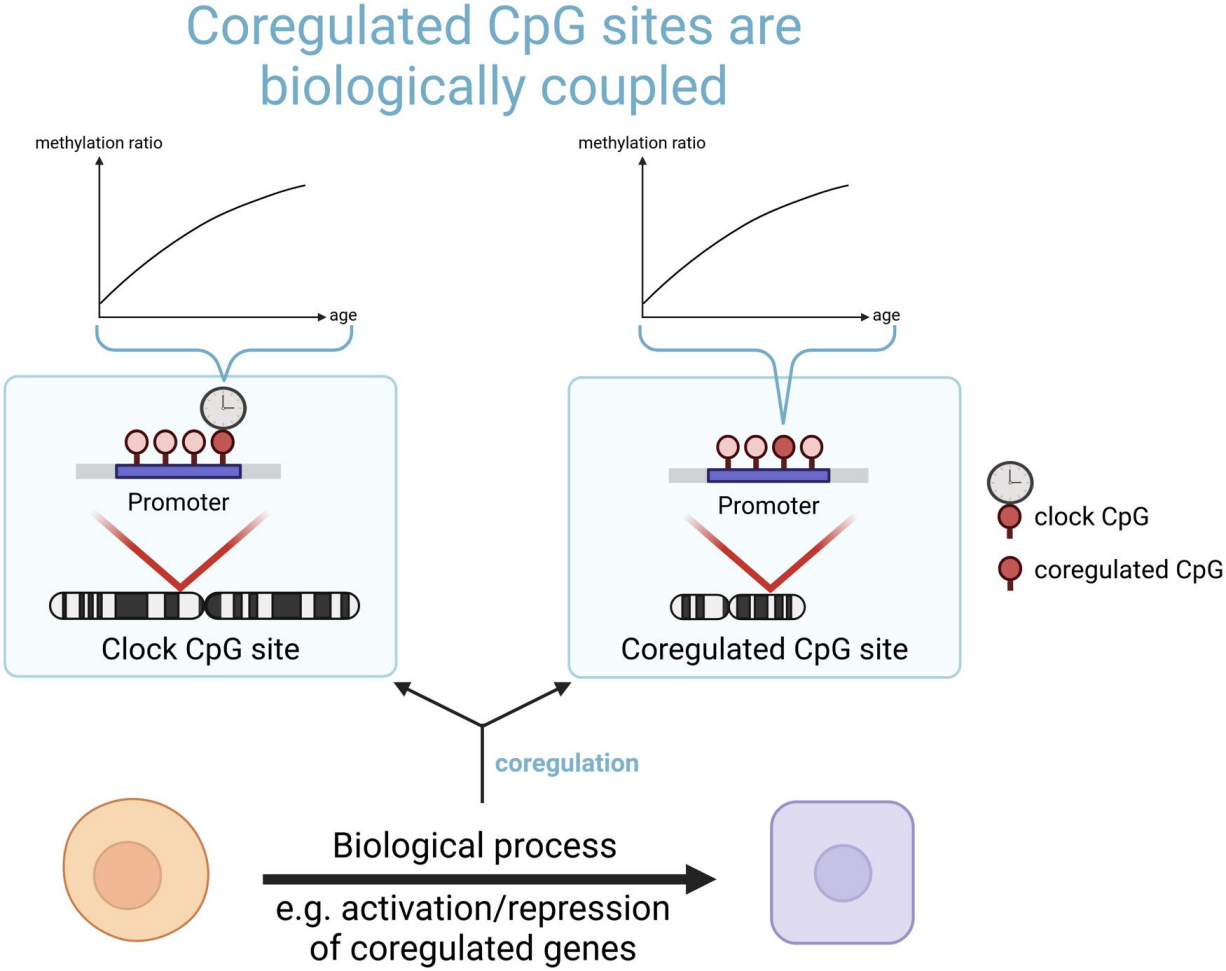
Biological processes can be distributed over long distances on the genome but coupled. Many biological processes are highly complex and can include genes that may be distantly located and even on different chromosomes. Although CpG sites are not neighboring, their methylation status can change in a coordinated way due to underlying co-regulated biological processes, leading to redundancy in DNAm patterning.

Although different authors have successfully created methylation clocks based on specific biological processes as e.g. cell division (Yang *et al.* 2016, Teschendorff 2020), the mechanisms that enable ticking of methylation clocks in general are still not fully understood. Consequently, several studies have recently been published that attempt to better understand the structure and properties of methylation clocks (Porter *et al.* 2021, Jonkman *et al.* 2022). One aspect that has been noted in such studies is the inherent redundancy in DNA methylation data. For example, it was shown in Porter *et al.* (2021) that many CpGs of a trained clock can be replaced by other CpGs without a noticeable loss in performance. As early as 2007, Horvath *et al.* described groups of coordinated CpGs with similar methylation status and named them “correlation networks,” and developed a corresponding analysis method, “WGCNA” (Langfelder and Horvath 2008). In a more recent study, Levine *et al.* clustered CpGs of different methylation clocks into functional “modules” based on their co-variation in different methylation datasets associated with aging, reprogramming, and cell culture passaging (Levine *et al.* 2022). Reasons for this correlation between CpGs can be the spatial proximity of CpGs as well as their functional coupling, caused by biological processes that affect different areas of the genome (Fig. 1). An example could be the binding of transcription factors, which usually have a high number of binding sites distributed over the genome and are influencing the methylation patterns surrounding their respective binding sites (Burger *et al.* 2013). For a recent review, see Seale *et al.* (2022). Consequently, recent studies (Porter *et al.* 2021, Jonkman *et al.* 2022) concluded that there was a significant degree of redundancy within the underlying data used for training of the clock.

An area that remains underinvestigated is the application of methylation clocks to single-cell data. Clocks that are applicable on a single-cell level could allow to disentangle the extent to which age prediction is driven by changes intrinsic to the cells or by changes in cell composition (Lappalainen and Greally 2017). Furthermore, a clock working on single cells could specify whether the ticking of the clock is based on a subset of cells or the population as a whole (Bell *et al.* 2019, Levine *et al.* 2022). The under-representation of single-cell clocks may partly be due to the fact that biological processes that take place over long time scales, such as the lifespan of an organism, are usually described with sufficient accuracy by bulk data. In addition, the amount of covered CpGs is usually small and varies strongly across individual cells, caused by the limited amount of sequencing reads assigned to a single cell in a typical single-cell methylation experiment (Mulqueen *et al.* 2018). This represents a fundamental obstacle to the application of methylation clocks to individual cells. Although the number of CpGs that usually define a clock is relatively small and in the range of a few hundred, there is still a high probability that many of the required CpGs are not covered in individual cells.

A recent study described a novel approach to predict the age of single cells using a probabilistic metric (Trapp *et al.* 2021). Although it allows predictions on the single-cell level, scAge does not make use of the possibilities of the established elastic net regression-based clock training. Moreover, it cannot be used together with already existing clocks, like the Hannum blood clock (Hannum *et al.* 2013) or the Horvath multi-tissue predictor (Horvath 2013). Here, we propose a different approach and present estiMAge, a framework that exploits the redundancy within DNA methylation data to approximate the values of missing clock CpGs. It extends the usual training algorithm for methylation clocks to single-cell data, making estiMAge applicable to newly developed clocks as well as already published ones. Our results show that estiMAge provides comparable results to scAge by applying it to single young and old hepatocytes and other benchmark single-cell methylation datasets. To demonstrate the robustness of estiMAge, we simulated increasing sparsity in single-cell data and evaluated the resulting prediction error. Overall, estiMAge represents a framework that allows the construction of single-cell methylation clocks based on the established elastic-net training approach and enables the application of single-cell versions of already established methylation clocks.

## 2 Methods

### 2.1 Design of the estiMAge clock algorithm

We aimed to develop an algorithm for the age prediction of single cells, which is as close as possible to the standard training process of DNA methylation clocks. For training the clock on bulk data, we followed the usual application of the elastic net regression using the glmnet package (Friedman *et al.* 2017). Although our training process of the clock on bulk data is quite analogous to the widely used training process (Horvath 2013), the main problem consists in the sparse coverage of single cells which makes the application of a trained clock to single cells not feasible at first glance. We aimed to solve this problem by substituting clock CpGs missing in individual single cells by sites showing a similar characteristic in the training data and being covered in the specific cell.

#### 2.1.1 Assigning similarity between CpG sites

In order to evaluate the similarity between CpGs, we used the Euclidean distance between CpGs, evaluated over the whole training set:

For each **CpG_i_** of the trained clock and for every **CpG_a_** located on the DNA methylation array we computed the Euclidean distance over the whole set of arrays contained in the training data:

$$\begin{array}{c} d\mathrm{istance}\left(CpG_{i},CpG_{a}\right)\;:=\sqrt{\left(\sum_{n}^{N}{\left(CpG_{i}\left(n\right)-CpG_{a}\left(n\right)\right)}^{2}\right)};\\ N=\mathrm{number}\;of\;\mathrm{arrays} \end{array}$$

In addition, we evaluated the performance of numerous further distance measures (Supplementary Fig. S2b). However, we found Euclidean distance showing the best performance in the datasets used for testing.

#### 2.1.2 Ordering of CpGs

For every CpG *C_i_* contained in the trained clock we evaluated the Euclidean distance of all other CpGs *C_j_* contained in the training set: dist_*i*_(*j*): = euclidean distance(*C_i_*, *C_j_*). Then we created a ranking based on ordering dist_*i*_ from lowest to highest distance, i.e. from the most similar CpG to the most distant one.

**Figure 2.**
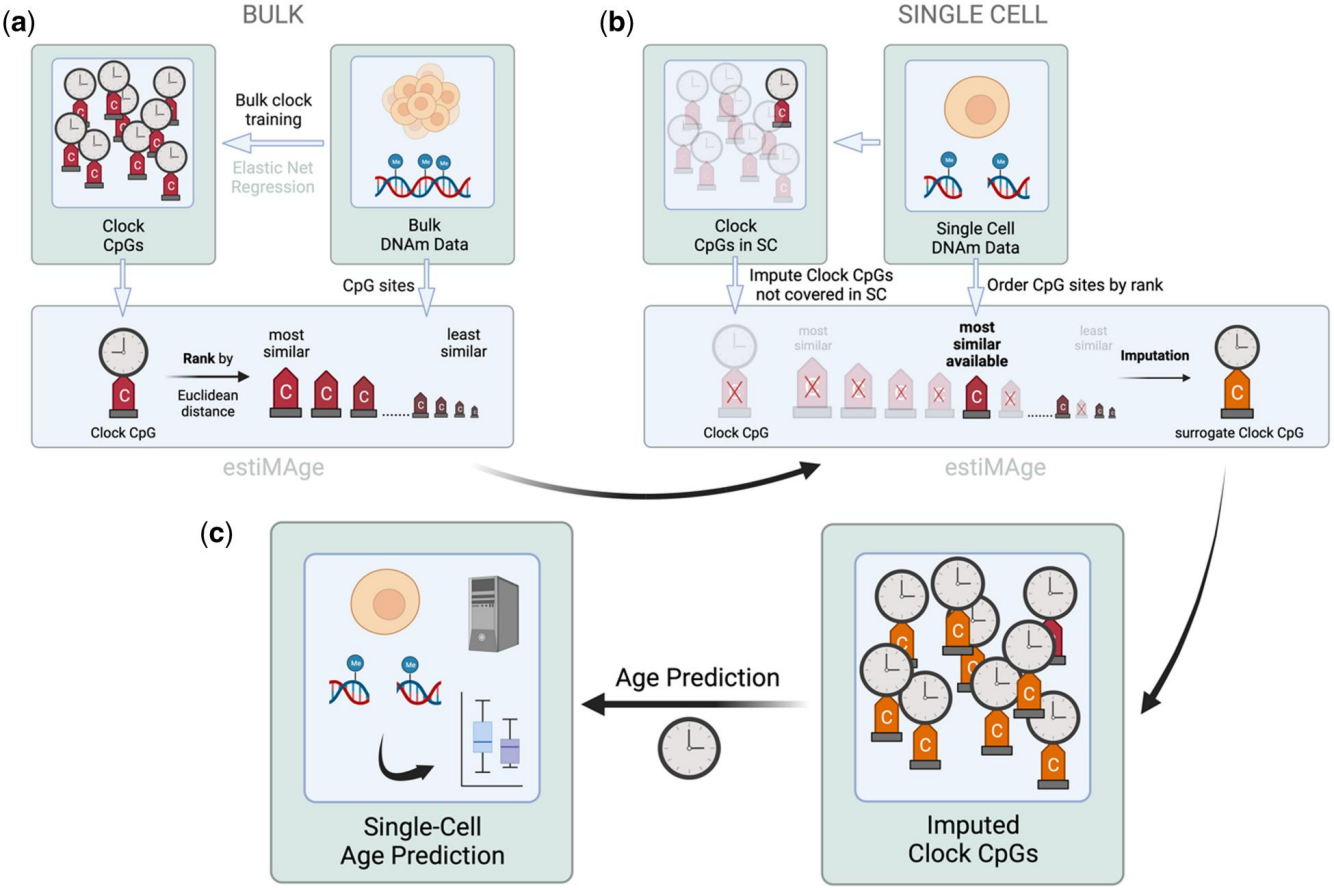
Basic workflow of single-cell prediction using estiMAge. (a) Bulk DNA methylation data are used to train a standard epigenetic clock by elastic net regression, resulting in a set of clock CpGs. estiMAge then ranks all other CpG sites covered in the bulk training data by their Euclidean distance to each clock CpG. (b) Due to the inherent sparsity of single-cell DNA methylation data, only a small fraction of clock CpGs will be covered in a cell. For each clock CpG that is not covered in a cell, estiMAge uses the rank of CpG sites produced in (a) and selects the most similar available CpG site to act as a surrogate for the missing clock CpG. (c) Using the imputed clock CpGs thus enables age predictions of the single cells with the standard bulk epigenetic clock.

To replace a clock CpG which was not covered in a specific cell, our framework used the ordered distances and traveled along the list, until the first CpG was found that was actually covered in the specific cell. The value of this CpG was used as a substitution for the clock CpG (Fig. 2).

## 3 Results

### 3.1 estiMAge trained on liver data predicts the age of single hepatocytes successfully

As an initial step, we trained a DNA methylation clock on a murine liver dataset from Thompson *et al.* (2018), which was sequenced based on the RRBS protocol. We then applied estiMAge to generate a single-cell version of the liver clock, which was subsequently used to predict single-cell hepatocytes (scHepatocytes) of different ages (Gravina *et al.* 2016). This dataset was already used by Trapp *et al.* (2021) as a central benchmark to evaluate the performance of the algorithm developed there. The dataset is well suited for this purpose, as the cells are related to precisely defined ages of 0, 4, and 26 months. The properties of the cells have been extensively described by Trapp *et al.* (2021). Thus, we only recapitulate the finding that the coverage of the individual cells is so low that the overlap between different cells is minimal, especially when iteratively overlapped. In accordance with Trapp *et al.* (2021), two outliers were removed from the complete dataset, and mouse embryonic fibroblasts (MEFs) were excluded from the prediction, resulting in a final dataset of 19 scHepatocytes. Prediction of the complete single-cell dataset including MEFs can be found in the Supplementary Materials (Supplementary Fig. S4).

In order to train bulk clocks in an unbiased way for further use as input for the construction of single-cell clocks, we performed a full cross-validation for all values of the parameter alpha between 0 and 1 in steps of 0.1. For every value of alpha, the function cv.glmnet which iterates through the penalty parameter lambda was used and the alpha value leading to the smallest cross-validation error (computed as the average over 100 repetitions) was selected. This resulted in values of 0.2 for the cross-validation on liver data (avg. cross-validation error: 1.74 months), of 0.3 for the cross-validation on blood data (cross-validation error: 1.21 months) and of 0.3 for the cross-validation on multiple tissues (cross-validation error: 3.41 months). These values of alpha were then applied for the training of bulk clocks on the respective set of samples. The liver clock trained with alpha of 0.2 contains 165 clock CpGs [based on *N* = 30 samples (C57BL/6J strain), Thompson *et al.* 2018], the tissue-specific blood clock 107 clock CpGs [based on *N* = 50 samples (C57BL/6J strain); Thompson *et al.* 2018), and the multi-tissue clock based on samples from liver, blood, muscle, kidney, lung, and adipose tissue 395 clock CpGs [based on *N* = 196 samples (C57BL/6J strain); Thompson *et al.* 2018]. The training data are identical to the data used by Trapp *et al.* (2021) to enable comparison of the results. The trained bulk clocks were then used as input for our estiMAge approach to create single-cell versions of these clocks.

**Figure 3.**
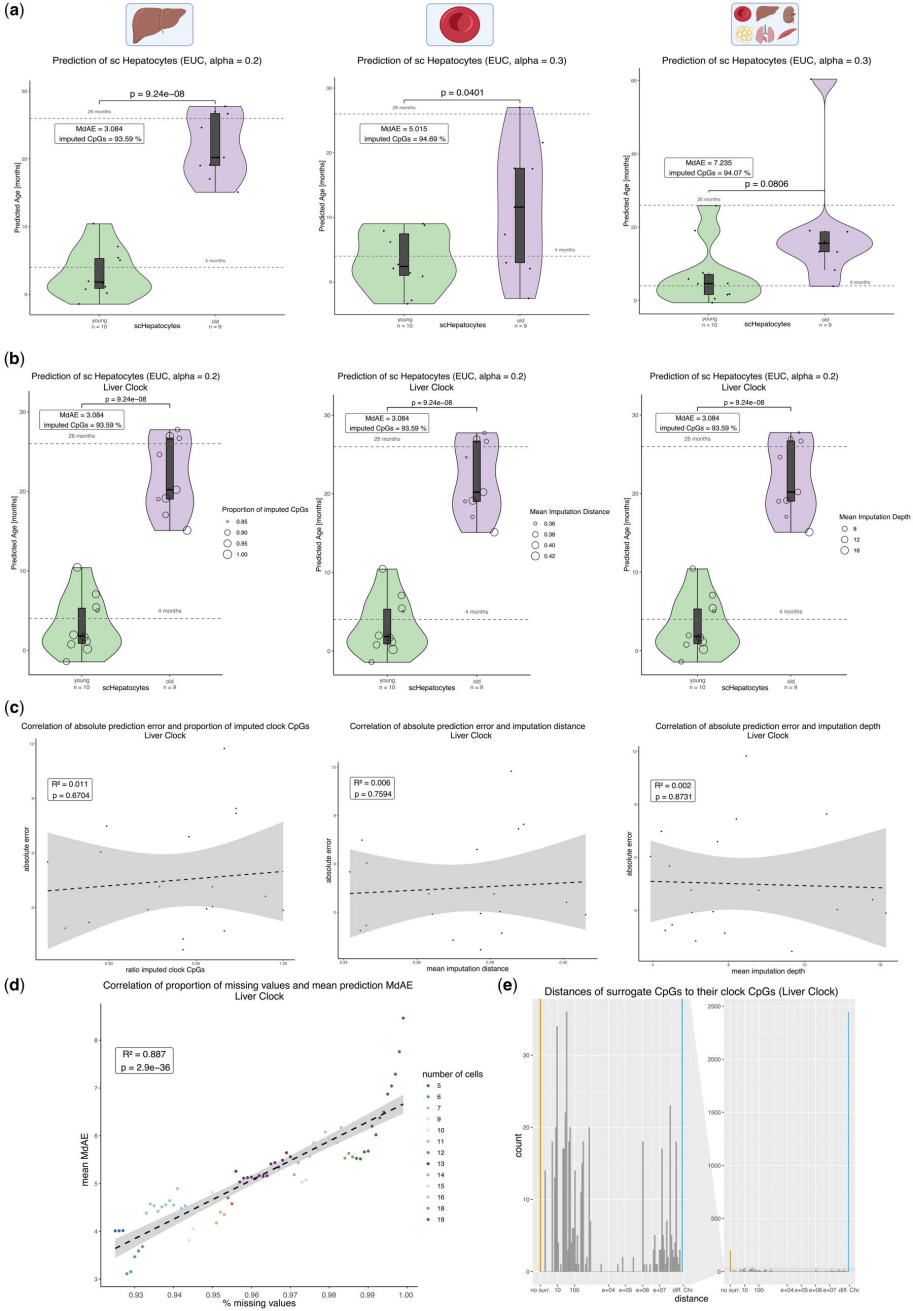
Prediction of single-cell hepatocytes using estiMAge. For statistical testing, two-tailed Welch’s *t*-test was used. (a) Epigenetic clocks trained on liver, blood, and multiple tissues (adipose, blood, kidney, liver, lung, muscle) were made single-cell competent by application of the estiMAge framework. Single-cell hepatocytes (*N* = 19 after outlier removal (Trapp *et al.* 2021), *N*_young_ = 10, *N*_old_ = 9) were predicted. Best overall prediction performance was achieved by the single-cell liver clock (alpha = 0.2, 165 clock CpGs), with a MdAE of 3.084 months. Old and young hepatocytes could be distinguished clearly (*P* = 9.24e−08). While the single-cell blood clock (alpha = 0.3, 107 clock CpGs) could also distinguish old and young hepatocytes (*P* = .0401), it could not predict the ages of the cells as well (MdAE = 5.015 months) as the other clocks. The single-cell multi-tissue clock (alpha = 0.3, 395 clock CpGs) resulted in poorer prediction quality than the blood clock (MdAE = 7.24 months) and failed to achieve a clear distinction of the groups (*P* = .0806). Median absolute deviations of predictions are shown in Supplementary Table S2. (b) Predictions of individual cells by the single-cell liver clock are sized according to proportion of imputed clock CpGs (left), mean imputation distance (middle) or mean imputation depth (right). There is no apparent trend linking smaller imputation ratios, distances, or depths to better prediction quality. (c) Plots of absolute prediction errors of the single cells against proportion of imputed clock CpGs (left), mean imputation distance (middle), or mean imputation depth (right). No clear correlation was detected. (d) Proportion of missing values in a single cell was progressively increased up to 99.9% by steps of 0.1%, and MdAEs for each ratio were evaluated. Starting point was the actual percentage of missing values in a cell, but minimally 92.5%. This guarantees an averaging over a minimum of five cells for each step, as only four cells fell below this number. Results are the average of *N* = 200 repetitions. Dots are colored according to the number of cells included in the calculation, with a minimum of five cells and a maximum of 19 cells. There is a significant (*P*-value = 2.9e−36) positive relationship between ratio of missing values and prediction MdAE. When increasing the ratio of missing CpGs from 99% to 99.9%, the slope increases strongly. Hence, while estiMAge apparently works better for cells with higher coverage, it can be used for single cells with a ratio of missing CpGs as high as 99% before the prediction becomes unreliable. (e) Spatial relation between clock CpGs and respective surrogate CpGs chosen by estiMAge, based on the prediction of scHepatocytes by the trained liver clock. 78.1% of surrogate CpGs are located on a different chromosome and another 4.2% on the same chromosome, but more than 1 Mb away.

The results of the prediction based on the single-cell liver clock (Fig. 3a, left) showed a clear separation between young hepatocytes of 4 months (predicted mean age = 3.23 months, predicted median age = 1.83 months) and old hepatocytes of 26 months age (predicted mean age = 21.85 months, predicted median age = 20.21 months). The median absolute prediction error (MdAE) of 3.08 months is slightly higher than the one reported for scAge of 2.1 months. The *P*-value for the separation between 4-month-old hepatocytes and 26-month-old hepatocytes was 9.24e−08.

In the case of the blood clock (Fig. 3a, middle), the prediction allowed a marginally significant distinction of the two groups (*P*-value = .0401). The prediction error (MdAE = 5.02 months) is greater than for the single-cell liver clock, but clearly better than the corresponding result for scAge based on blood, which is 10 months. Finally, the trained multi-tissue clock cannot clearly distinguish young and old hepatocytes (*P*-value = .0806) and leads to an age prediction quality (MdAE = 7.24 months) worse than the blood trained clock and also worse than the corresponding result for scAge trained on multiple tissues of 4.5 months. The poor performance of the multi-tissue clock on single-cell data is not surprising given relatively high cross-validation error of the underlying bulk clock.

### 3.2 estiMAge prediction is robust against imputation depth and imputation distance

Next, we analyzed the relation of the prediction quality of the single-cell liver clock to different properties of the imputation process (Fig. 3b and c, corresponding results for the other clocks are shown in Supplementary Fig. S3). Figure 3b shows the proportion of clock CpGs that had to be imputed (left), the mean “imputation distance” (middle) and the mean “imputation depth” (right) for each cell. The imputation distance corresponds to the Euclidean distance of a clock CpG to its surrogate CpG, while the imputation depth corresponds to the number of steps that had to be taken by the algorithm until a surrogate value was found. Both are used as measures for similarity of the surrogate CpG to the clock CpG and are dependent on the coverage of the single cells to predict. The result of this analysis showed that no significant correlation of these parameters and the prediction quality was apparent (Fig. 3b), indicating robustness of the algorithm against variation in the CpG coverage within the cells. The same holds true when plotting the parameters against the absolute prediction errors (predicted versus actual age) of a single cell (*P*-values ≫ .05; Fig. 3c). To further test the dependence of the prediction quality on the sequencing coverage, the amounts of missing values in single hepatocytes was progressively increased from the actual minimal value in a cell of 92.5% up to 99.9% by artificially introducing missing values and evaluation of the resulting prediction quality (200 repetitions, increment in steps of 0.1) (Fig. 3d). The median absolute prediction error (MdAE) increased modestly up to 99% of missing values from 3.5 to about 6, but rose strongly when increasing the ratio of missing CpGs from 99% to 99.9%. This indicates that, while estiMAge works better with higher coverage, it can be used for single cells with a ratio of missing CpGs as high as 99% before the prediction becomes unreliable. Remarkably, the value of 92.5% uncovered CpGs corresponds to a number of only 95 000 CpGs covered in a cell and overlapping with the training data, making for an overall impressive accuracy when comparing against scAge, where a stable prediction was only achieved for a coverage exceeding 500 000 CpGs (Trapp *et al.* 2021).

### 3.3 Surrogate CpGs are distributed over the genome

Next, we analyzed the spatial relation between clock CpGs and respective surrogate CpGs chosen by estiMAge, based on the prediction of scHepatocytes by the trained liver clock (Fig. 3e). We found that a large majority of surrogate CpGs (78.1%) was located on a different chromosome and another 4.2% on the same chromosome, but more than 1 Mb away. Of the remaining surrogate CpGs, nearly all (11.1% of all surrogate CpGs) were located within a range of 1000 bp to the respective clock CpG. This underlines that, for the most part, common biological processes are driving the similarity between surrogate and clock CpGs. The observed proportion of surrogate CpGs located within 1 kb of a clock CpG likely represent short-range synchronization of methylation dynamics, as these CpGs are located in the same genomic compartment, e.g. CGI, promoter, enhancer, etc. A further characterization of surrogate CpGs is described in Supplementary Text S1 and Supplementary Tables S3–S5.

### 3.4 Application of estiMAge to existing epigenetic clocks

**Figure 4.**
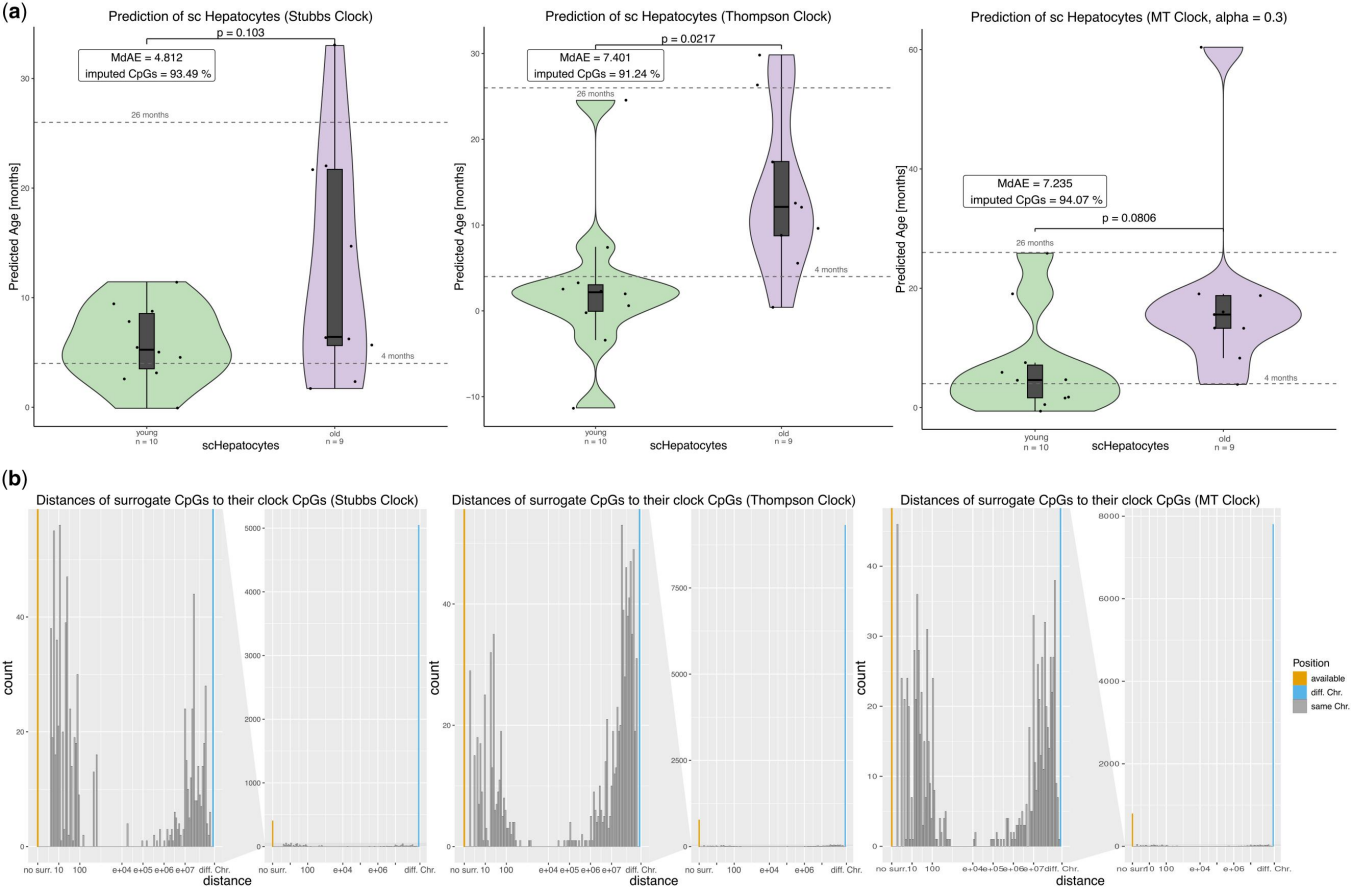
Prediction of single-cell hepatocytes with single-cell versions of pre-existing murine multi-tissue clocks. Single-cell versions of Stubbs and Thompson multi-tissue Clocks were used to predict the ages of scHepatocytes (Gravina *et al.* 2016). Results of the age prediction with the self-trained multi-tissue clock (alpha = 0.3) were included for comparison. For statistical testing, two-tailed Welch’s *t*-test was used. (a) Prediction of young (4 months old, *n*_young_ = 10) and old (26 months old, *n*_old_ = 9) single-cell hepatocytes (Gravina *et al.* 2016). The Stubbs Clock showed the smallest MdAE (MdAE: 4.81 m), compared to the Thompson clock (MdAE: 7.4 m) and the self-trained multi-tissue clock (MdAE: 7.24 m). However, the Thompson clock could achieve significant separation of young and old hepatocytes (*P* = .0217). (b) Distances of surrogate CpGs to respective clock CpGs. Proportions observed were similar like for single-cell liver clock (Fig. 3e). The trends are similar for all three clocks. Over 80% of surrogates were located on a different chromosome (Stubbs: 80.7%, Thompson: 84.3%, self-trained multi-tissue clock: 85.6%). Around 10% were on the same chromosome (Stubbs: 12.7%, Thompson: 8.7%, self-trained multi-tissue clock: 8.4%), among which similar parts were located either within 1 kb or further than 1 Mb away.

A basic strength of the proposed framework is the possibility to generate single-cell versions of any existing epigenetic clock, as long as access to training data is provided. To demonstrate a proof of this concept, the estiMAge framework was used to generate single-cell versions of well-established murine multi-tissue clocks (Stubbs *et al.* 2017, Thompson *et al.* 2018). The single-cell versions of these clocks were then used for age prediction of scHepatocytes and the results were compared to the self-trained multi-tissue clock (alpha = 0.3) (Fig. 4). The Stubbs clock showed the best performance (MdAE = 4.81 months, *P* = .103) whereas the self-trained multi-tissue clock (MdAE = 7.24 months, *P* = .0806) and the Thompson clock (MdAE = 7.40 months, *P* = .0217) resulted in a poorer prediction performance. However, the Thompson clock showed a significant separation of the young and old hepatocytes (*P* = .0217) (Fig. 4a). All three clocks underestimate the age of 26 months old hepatocytes, whereas cells of 4 months of age are predicted relatively precisely. The distances of the surrogate to the respective clock CpGs show a similar distribution like for the self-trained single-cell liver clock, confirming that surrogate CpGs are chosen based on their biological relation rather than their spatial proximity (Fig. 4b).

### 3.5 Application of estiMAge to additional benchmark datasets

As real single-cell methylation datasets are still rare, for sake of comparison we have collected all available datasets discussed in Trapp *et al.* and performed predictions using estiMAge. This contains mouse gastrulation data, ES cells grown in 2i/serum medium and muscle stem cells. For these datasets, a precise numerical age is missing, although basic assumptions about the expected cell ages exist. Our computations demonstrate results similar to those found by Trapp *et al.* in all cases and are explained in detail in Supplementary Text S1 and Supplementary Fig. S1.

## 4 Discussion

Here, we introduce estiMAge, a tool for age prediction of single-cell methylation data. Our results demonstrate that estiMAge can be used to generate single-cell versions of existing epigenetic clocks, allowing for flexibility in the application of our framework. We also showed that estiMAge, applied to a liver-specific epigenetic clock, can predict the age of single hepatocytes with a remarkable prediction accuracy. Within the given range, the quality of the prediction was shown to be independent from the proportion of imputed clock CpGs, as well as from the imputation distance and depth. In contrast to a previously published approach (Trapp *et al.* 2021), estiMAge represents a framework to construct genuine DNA methylation clocks. The principal idea is to exploit the redundancy in DNA methylation data, to substitute missing CpGs required by the trained clock by similar ones. We show that, for different biological systems like hepatocytes or cultured ES cells the accuracy of prediction is comparable to the results shown in (Trapp *et al.* 2021). For hepatocyte data, the accuracy of the clocks trained on liver, as well as on multiple tissues based on the median absolute error is similar to the published results, whereas for the clock trained on blood it is clearly better.

By simulating cells with a varying amount of coverage, we explore the limits of our approach. We show that up to a proportion of 99% missing CpGs the predictions are still robust. This makes our tool applicable even for high-throughput DNA single-cell methylation analyses, for which values of average coverage of about 1% for individual cells have been reported (Mulqueen *et al.* 2018, Solé-Boldo *et al.* 2022). By using estiMAge to impute missing CpG sites in single cells, we provide a proof of concept and thus enable analyses that are normally not feasible due to the sparsity of single-cell data.

We consider the main advantage of this type of single-cell clock that it represents a “genuine” DNA methylation clock in the sense of the clocks which have been developed during the last decade. This is not only of importance for the sake of compatibility with known approaches but also allows to exploit the existing advantages of the usual clock training algorithm. Adjusting the value of the parameter alpha for the training influences the composition of the elastic net regression: using alpha closer to 1 results in a bigger influence of Lasso regression, therefore eliminating more CpGs from the model. Consequently, by varying the parameter alpha, the number of CpGs defining the trained clock can be controlled and the amount of redundancy contained in the clock can be adjusted. This enables some flexibility to tune the clock for specific datasets or purposes.

It has been shown that DNA methylation clocks can be influenced by age-dependent changes in cell-type composition of the corresponding tissue (Jonkman *et al.* 2022, Gorelov *et al.* 2024). While the elastic-net based algorithm applied for clock training penalizes CpGs which are strongly influenced by age-independent changes of methylation, future studies should test the effects of cell-type composition (e.g. immune cells) before applying estiMAge (or similar clocks) to bulk data. In general, the quality of the used bulk clock will influence the prediction on the single-cell level. Although using a bulk clock trained on sorted cell types would be ideal, the good performance of blood-trained estiMAge on hepatocytes suggests a considerable robustness of our approach.

Based on this, our proposed approach opens the possibility to develop virtually any existing DNA methylation clock into a single-cell clock, without changing the clock as such, as long as the training data of the clock is available. We have provided proof of concept for this by generating single-cell versions of two well-established murine multi-tissue clocks (Stubbs *et al.* 2017, Thompson *et al.* 2018). For other purposes and species, the application of estiMAge to other well-established clocks like the multi-tissue age predictor (Horvath 2013), the Hannum blood clock (Hannum *et al.* 2013), EpiTOC2 (Teschendorff 2020) or even second-generation clocks like the PhenoAge (Levine *et al.* 2018) or the GrimAge clock (Lu *et al.* 2019) can be explored. The platform Biolearn collects information of a substantial number of published epigenetic clocks and thus provides an attractive starting point (Ying *et al.* 2024b). By creating single-cell versions of these clocks using estiMAge and integrating them in such platforms, a simple application of single-cell DNAm age estimation would be possible.

To support the integration of epigenetic clocks into clinical practice as a biomarker of aging, a single-cell approach by estiMAge could help to overcome major obstacles. As illustrated in a recent work, a range of criteria are employed to assess biomarkers. Single-cell data could facilitate a deeper understanding of the mechanistic basis of aging and enhance the generalizability of epigenetic clocks, which is currently constrained by variations of cell-type compositions, among other factors (Moqri *et al.* 2023). Furthermore, a direct single-cell application could not only contribute to understanding, but also has the potential to provide valuable information. EstiMAge would allow to estimate how much the methylation ages of individual cells in a bulk sample are varying and whether the amount of variation is increasing with age or due to underlying diseases. This would enable the prediction of a methylation age on the single-cell level as a biomarker of clinical relevance (Moqri *et al.* 2023) or could be incorporated in possible omics-biomarkers (Moqri *et al.* 2024a).

### 4.1 Limitations

An important limitation is the current shortage of scMeth-seq data. This makes it difficult to test our approach extensively on a practical level to find relevant weaknesses and to improve the algorithm accordingly. Computations with simulated data might be an alternative, but cannot completely substitute for practical data. However, we expect this situation to improve, given the increasing availability of scMeth-seq protocols. Secondly, if self-trained clocks are aimed at, the training algorithm could be further improved. A cross-validation scheme for training, which combines training on a portion of bulk data with prediction on a portion of single-cell data would be an option. In this case, the amount of available scMeth-seq data has to be high enough. Such a training scheme could also help to address potential confounding factors of bulk clocks, including their sensitivity to age-related changes in cell-type proportions. However, if established clocks like the Hannum blood clock are used together with estiMAge, this issue does not play a role. In this situation, a mandatory prerequisite for using estiMAge is access to the training data of this clock, which is usually given, but limited in some cases.

## Supplementary Material

vbaf005_Supplementary_Data

## Data Availability

To train single and multi-tissue clocks we used publicly available datasets from the GEO database (GSE120137). To apply estiMAge to single-cell data, we used datasets GSE121690, GSE68642, GSE121364 and SRA344045. More details about the datasets can be found in Supplementary Table S1.

## Program availability

The source code and instructions for usage of estiMAge are available at https://github.com/DivEpigenetics/estiMAge.
