# Supplementary material for: estiMAge: development of a DNA methylation clock to estimate the methylation age of single cells
Source: Bioinform Adv. 2025 Jan 16;5(1):vbaf005. doi: 10.1093/bioadv/vbaf005 (PMC11769677; doi:10.1093/bioadv/vbaf005)
Supplement: vbaf005_Supplementary_Data [file vbaf005_supplementary_data.zip › FigureS3e.pdf]

e

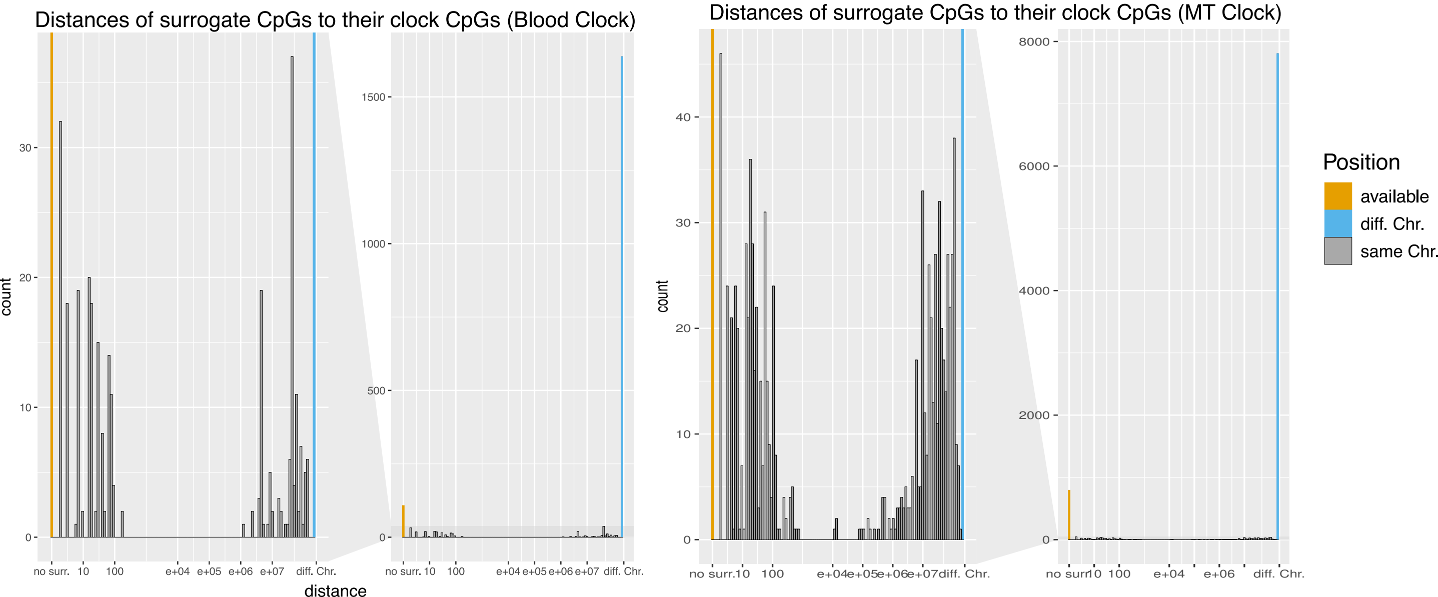

Distances of surrogate CpGs to their clock CpGs (Blood Clock)
count
distance
no surr.
10
100
e+04
e+05
e+06
e+07
diff. Chr.
0
10
20
30
500
1000
1500
Distances of surrogate CpGs to their clock CpGs (MT Clock)
40
2000
4000
6000
8000
Position
available
diff. Chr.
same Chr.
