# Supplementary material for: estiMAge: development of a DNA methylation clock to estimate the methylation age of single cells
Source: Bioinform Adv. 2025 Jan 16;5(1):vbaf005. doi: 10.1093/bioadv/vbaf005 (PMC11769677; doi:10.1093/bioadv/vbaf005)
Supplement: vbaf005_Supplementary_Data [file vbaf005_supplementary_data.zip › estiMAge_sassmannshausen_supplement_cc.docx]

**Supplementary Text**

**1. Application of estiMAge to other single-cell datasets**

For benchmarking our single-cell epigenetic clock framework, we have applied estiMAge to additional available single-cell methylation datasets also predicted using scAge (Trapp et al., 2021).

**estiMAge can distinguish embryonic stem cells derived from different growth conditions**

The first dataset used comprises 81 mouse embryonic stem cells (mESCs) cultivated in either medium supplemented with serum (n_Serum_ = 16) or serum-free medium supplemented with two inhibitors (2i medium; n_2i_ = 65). Although there is no quantitative age associated with these cells, the different cultivation media are known to lead to a measurable age difference. While cells cultivated in both media should be found to have low ages, culturing mESCs in 2i medium has been shown to lead to epigenetic profiles concordant with migratory primordial germ cells (Ficz et al., 2013). Thus, the epigenetic age of 2i-cultured cells should be predicted to be greater than that of those cultured in serum. Prediction was performed with all three previously described single-cell epigenetic clocks (trained on data from liver, blood, or multi-tissue) and led to a clear separation of serum- and 2i-cultured cells, with the latter being predicted significantly older than the former (liver clock: p-value = 2.52 e-11, blood clock: p-value = 9.69 e-09, multitissue clock: p-value = 4.16 e-08) (Figure S1a). These results are also in accordance with the results achieved by scAge (Trapp et al., 2021).

**estiMAge can distinguish different mouse gastrulation stages**

Next, a data set consisting of single cells isolated from murine embryos of different embryonic stages (E4.5, E5.5, E6.5 and E7.5) was used for prediction (Argelaguet et al., 2019; Figure S1b). Cells with a coverage of up to 500,000 CpGs sites were excluded from the prediction, resulting in a final dataset of 503 single cells. We expected to see a decline in the predicted ages from E4.5 to E7.5, since there is evidence of a rejuvenation event during embryogenesis (Kerepesi et al., 2021). This decline in the epigenetic age of the cells was captured by all three epigenetic clocks. Distinction of the four stages was most significant for the liver clock, with the p-values being significant for E4.5 against E5.5 and E5.5 against E6.5. The blood clock yielded similar results but could significantly distinguish E6.5 from E7.5 instead of E5.5 from E6.5. The multi-tissue clock showed a similar performance and could significantly distinguish cells from E4.5 against E5.5 and E5.5 against E6.5. As there are no quantitative ages annotated, the prediction performance can only be evaluated by the clock’s ability to capture the overall rejuvenation trend, which was largely achieved.

**estiMAge predicts attenuated aging of muscle stem cells**

Lastly, estiMAge was tested on murine muscle stem cells (muSCs) obtained from three young (1.5 months) and three old (26 months) mice (Hernando-Herraez et al., 2019). In accordance with the original study and Trapp et al. (2021), only cells with a minimum coverage of 500,000 CpG sites were kept for the analysis, resulting in a final dataset of 205 muSCs (n_young_ = 89, n_old_ = 116). Here, we expected to capture attenuated epigenetic aging observed for muscle stem cells in the original study (Hernando-Herraez et al., 2019). For this, we used a single-cell version of a clock trained on muscle instead of liver tissue to predict the single cells (based on N = 30 samples, alpha = 0.1). While only the multi-tissue clock detects a significant difference between young and old muSCs (p = 0.000156) (Figure S1c), both the muscle and blood clock result in only a marginally insignificant distinction (p_muscle_ = 0.0715, p_blood_ = 0.0709). Smallest MdAE was achieved by the single-cell clock based on muscle tissue (MdAE = 9.503 months), followed by the multi-tissue- (MdAE = 9.504 months) and the blood (MdAE = 9.972 months) based single cell clocks. All three clocks predict the ages of the older muSCs to be higher than that of the younger cells (mean_old,muscle_ = 4.4 months, mean_young,muscle_ = 2.9 months; mean_old,blood_ = 9.7 months, mean_young,blood_ = 8.2 months; mean_old,multi-tissue_ = 15.8 months, mean_young,multi-tissue_ = 10.8 months), but predict the old muSCs far younger than their chronological age of 26 months, confirming the attenuated epigenetic aging observed in the original study and by scAge prediction (Hernando-Herraez et al., 2019; Trapp et al., 2021). Median absolute deviations of predictions are shown in Table S2.

Taken together, application of estiMAge on all available single-cell benchmark datasets confirmed trends also captured by scAge (Trapp et al., 2021). Thus, we conclude that estiMAge can be readily used for prediction of any available single-cell methylation dataset.

**2. Characterization of surrogate CpGs**

The basic assumption of the design of estiMAge is that groups of CpGs follow a common, biologically determined dynamic over time, even when they are not located in proximal distance in the genome. As shown in Figure 3e the majority of found surrogate CpGs indeed is located on another chromosome than the original CpG. Using the trained liver clock as an example, we have analyzed the relation of clock CpGs and their surrogates for the chromHmm segmentation of liver tissue available at the UCSC genome server (https://genome.ucsc.edu). Table S3 shows that the 1^st^ surrogate of a CpG indeed has a high probability to be located in the same genomic context as the clock CpG to be replaced. Interestingly, in the case of generalized segments this probability is approximately stable up to the 10^th^ surrogate.

We further asked whether the distribution in different chromHmm segments is similar for the original set of clock CpGs and the first surrogates. As is shown in Table S4 the overall distribution is rather similar. This is further refined into specific changes between individual segments (Table S5). Interestingly, clock CpGs located in bivalent promoter segments often have surrogates in repressed regions or in active promoters, which are the segments bivalent promoters can turn into. An exemplaric biological process affecting the methylation level of CpGs in a similar way over long genomic distances is epigenetic drift, which is pronounced especially in PRC2 repressed regions. Interestingly, methylation clocks often contain subsets of CpGs located in PCR2 bound regions (Horvath and Raj, 2018). For the trained liver clock, for the 16 clock CpGs located in PRC2 repressed regions 6 had a first surrogate in this segment (Hc-P) as well and another 4 had the first surrogate in bivalent promoters, which are also bound by PRC2 (Table S5). The remaining 6 CpGs are located in enhancers and promoters. This underlines the tendency for CpGs to have surrogates which are located in PRC2 repressed regions as well. We further investigated functionally active segments like active promoters and transcribed regions. Although there is a clear tendency for surrogate CpGs to be placed in the same type of segment a specific relation for individual CpGs is difficult to characterize. An example is the clock CpG chr17:46629141 located in the promoter of the signaling protein Ptk7 which has a first surrogate in the promoter of Scrt2 (chr2:152082171) which is a transcriptional regulator. Although there might be a biological relation a direct connection is not described so far for these genes.

**Supplementary discussion**

**A note on a recently published PRC2-AgeIndex**

Interestingly, a recent paper (Moqri et al., 2024b) introduces an assay agnostic
approach which exploits the general trend of methylation increase in PRC2 repressed regions over time, which have been observed to be contained in different established methylation clocks. Based on this the authors define a "PRC2-AgeIndex", which serves as a biomarker of aging by evaluating the genome wide average methylation level in these regions of a subject. The authors apply their approach to the dataset of single hepatocytes from Gravina et al. as well and show that the resulting measure, although not assigning a concrete age to the cells, enables a statistically significant separation between young (4 m) and old (26 m) hepatocytes. From our point of view, this approach has some similarity to the idea which estiMAge is based on as it assumes that the general trend of methylation change over time is similar between CpGs in PRC2 repressed regions to allow defining an overall measure, no matter which individual CpGs are covered in a specific cell and where on the genome these CpGs are located. We consider the main difference to the PRC2-AgeIndex that estiMAge takes arbitrary genomic regions into account, predicts a concrete age and can be applied to basically any existing clock, whereas the PRC2-AgeIndex focuses on a specifictype of genomic region and aims at an relatively easy, but robust biomarker.

**Figure S1.**

**Prediction of other available benchmark single-cell datasets with estiMAge.**

Single-cell versions of liver, blood and multi-tissue clocks were used to predict the ages of three available single-cell datasets. a) Prediction of mESCs cultivated in either serum (n_Serum_ = 16 or medium supplemented with 2 inhibitors (2i medium); n_2i_ = 65). Significant distinction was achieved by all three clocks (p_Liverclock_ = 2.53 e-11, p_Bloodclock_ = 9.69 e-09, p_Multitissueclock_ = 4.16 e-08). Separation was clearest for the single-cell liver clock, followed by blood and multi-tissue based clocks. Cells cultured in 2i medium were predicted to be older than cells cultured in serum (liver clock: mean_serum_ = 3.68 months, mean_2i_ = 14.20 months; blood clock: mean_serum_ = -0.76 months, mean_2i_ = 5.20 months; multi-tissue clock: mean_serum_ = 12.17 months, mean_2i_ = 31.86 months). For statistical testing, two-tailed Welch’s t-test was used. b) Prediction of single cells obtained from mouse embryos of different developmental stages (E4.5-E7.5) (N = 503 cells after coverage filtering >500,000 CpGs). For statistical testing, two-tailed Welch’s t-test with Bonferroni correction was used based on the R function p-adjust with default settings (n = 3, corresponding to the number of p-values to be adjusted). All three clocks capture decrease in epigenetic age during embryo development. Liver clock could significantly distinguish E4.5 from E5.5 (p-value = 1.02 e-16) and E5.5 from E6.5 (p-value = 8.79 e-06). Blood clock could separate E4.5 from E5.5 (p-value = 2.97 e-06) and E6.5 from E7.5 (p-value = 0.0141). Multi-tissue clock could significantly separate cells from E4.5 and E5.5 (p-value = 2.69 e-14) and E5.5 from E6.5 (p-value = 0.019). c) Prediction of muSCs obtained from three young (1.5 months) and three old (26 months) mice (Hernando-Herraez et al., 2019) (N = 205 muSCs after coverage filtering >500,000 CpGs; n_young_ = 89, n_old_ = 116). A single-cell version of a muscle clock (based on N = 30 samples, alpha = 0.1) was used instead of the single-cell liver clock. Of the three clocks used only the multi-tissue clock detects a significant difference between young and old muSCs (p_muscle_ = 0.0715, p_blood_ = 0.0709, p_multitissue_ = 0.000156). Prediction was most accurate for the muscle clock (MdAE = 9.503 months), followed by the multi-tissue (MdAE = 9.504 months)-based and the blood (MdAE = 9.972 months) single cell clocks. All three clocks predict the ages of the older muSCs to be higher than that of the younger cells (mean_old,muscle_ = 4.4 months, mean_young,muscle_ = 2.9 months; mean_old,blood_ = 9.7 months, mean_young,blood_ = 8.2 months; mean_old,multi-tissue_ = 15.8 months, mean_young,multi-tissue_ = 10.8 months), but predict the old muSCs far younger than their chronological age of 26 months.

**Figure S2.**

**Evaluation of prediction performance for different alphas and distance metrics.**

a) Prediction performance of single-cell liver clock applied to scHepatocytes (Gravina et al., 2016) for different values of alpha. Root-mean squared error (RMSE), mean absolute error (MAE) and median absolute error (MdAEs) are depicted as quality measures. Final comparisons are done with MdAEs, in line with analyses by Trapp et al. (2021). Dotted line indicates alpha for which prediction errors were minimal. b) Prediction performance of single-cell liver clock applied to scHepatocytes (Gravina et al., 2016) for different distance metrics at different alphas. The following distance metrics were tested to generate the order of potential clock CpG substitutes: Euclidean (EUC), absolute correlation distance (ABS), Canberra distance (CAN), correlation distance (COR), Hamming distance (HAM), Jaccard distance (JAC), Manhattan distance (MAN), Maximum distance (MAX). Absolute minimum of all distance measures and alphas is indicated with an arrow. Evidently, Euclidean distance outperforms other distance measures, with the Manhattan distance following after. Consequently, all further analyses were made on the basis of Euclidean distances. c) MdAE for single-cell blood, liver and multi-tissue epigenetic clocks at different alphas. Arrows indicate the respective minima.

**Figure S3.**

**Prediction of single-cell hepatocytes with single-cell blood- and multi-tissue clocks, evaluation of clock features.**

a) Predictions of individual cells by the single-cell blood clock are sized according to proportion of imputed clock CpGs (left), mean imputation distance (middle) or mean imputation depth (right). There is no apparent trend linking smaller imputation ratios, distances or depths to better prediction quality. b) Predictions of individual cells by the single-cell multi-tissue clock are sized according to proportion of imputed clock CpGs (left), mean imputation distance (middle) or mean imputation depth (right). There is no apparent trend linking smaller imputation ratios, distances or depths to better prediction quality. c) Plots of absolute prediction errors of the scHepatocytes by the single-cell blood clock against proportion of imputed clock CpGs (left), mean imputation distance (middle) or mean imputation depth (right). Negative correlation was detected for all three cases. However, this was insignificant. d) Plots of absolute prediction errors of the scHepatocytes by the single-cell multi-tissue clock against proportion of imputed clock CpGs (left), mean imputation distance (middle) or mean imputation depth (right). No clear correlation was detected for all three cases. e) Distances of surrogate CpGs to actual clock CpGs. In the case of the blood clock, 5.31 % of clock CpGs were covered (available, orange), while 80.52 % of the surrogate CpGs localized to a different chromosome than the clock CpG (blue). From the remaining 14.16 % of surrogates that were located on the same chromosome as the clock CpG (grey), 58.33 % were located within 1000 bases distance to the clock CpG, suggesting spatial relation to the clock CpG. Another 41.66 % were located further than 1,000,000 bases from the clock CpG, where a spatial relation can practically be ruled out. Similarly, for the multi-tissue clock, 5.93 % of clock CpGs were covered (available, orange), while 84.66 % of the surrogate CpGs localized to a different chromosome than the clock CpG (blue). From the remaining 9.41 % of surrogates that were located on the same chromosome as the clock CpG (grey), 48.58 % were located within 1000 bases distance to the clock CpG, suggesting spatial relation to the clock CpG. Another 48.87 % were located further than 1,000,000 bases from the clock CpG. Collectively, this demonstrates that there is no clear bias for spatially close CpGs to be chosen to act as surrogates.

**Figure S4**

**Prediction of single-cell hepatocytes and MEFs with single-cell liver, blood, and multi-tissue clocks.**

For statistical testing, two-tailed Welch’s t-test with Bonferroni correction was used. a) Prediction of all single-cell hepatocytes including outliers and mouse embryonic fibroblasts (MEFs) (N_mefs_ = 5, N_hep_young_ = 11, N_hep_old_ = 10). None of the clocks could separate MEFs from young hepatocytes. Only the liver clock could clearly distinguish young from old hepatocytes (p-value = 1.65 e-06). Best prediction accuracy was achieved for liver clock (MdAE = 3.483 m), followed by the blood clock (MdAE = 4.959 m) and the multi-tissue clock (MdAE = 8.734 m). b) Prediction of all scHepatocytes including mouse embryonic fibroblasts (MEFs). Two outliers were removed (N_mefs_ = 5, N_young_ = 10, N_old_ = 9). None of the clocks could separate MEFs from young hepatocytes. Only the liver clock could clearly distinguish young from old hepatocytes (p-value = 1.85 e-07). Best prediction accuracy was achieved for liver clock (MdAE = 3.14 m), followed by the blood clock (MdAE = 4.846 m) and the multi-tissue clock (MdAE = 7.365 m).

**Table S1: Used datasets.** Overview of used data sets (bulk vs single-cell), with composition and accession numbers.

| **Author** | **Accession** | **Number of samples** | **Species** | **Tissue** | **Experiment** |
| --- | --- | --- | --- | --- | --- |
| Thompson et al. | GSE120037 | 196 (C57BL/6J strain) (all strains: 386) | mice | adipose blood liver lung kidney muscle | Illumina HiSeq 2500 (Mus musculus) Illumina HiSeq 4000 (Mus musculus) |
| Angermüller et al. | GSE68642 | 81 | mice | embryonic stem cells | bisulfite-sequencing |
| Gravina et al. | SRA344045 | 26 | mice | muscle embryonic fibroblasts Hepatocytes | WGBS |
| Argelaguet et. al | GSE121690 | 504 | mice | embryos of different gastrulation stages (E4.5, E5.5, E6.5, E7.5) | scNMT-seq (single-cell Nucleosome, Methylome and Transcriptome sequencing) |
| Hernando-Herraez et. al | GSE121364 | 205 | mice | muscle stem cells (muSCs) | parallel single-cell DNA methylation and transcriptome sequencing (scM&T-seq) |

**Table S2:** Variance of the predictions measured by median absolute deviation (MAD) for different clock predictions. The first value in a cell is MAD, second value is MdAE.

|  | **Liver clock** | **Blood clock** | **Multitissue clock** | **Stubbs** | **Thompson** | **Muscle clock** |
| --- | --- | --- | --- | --- | --- | --- |
| **Hepatocytes** | 4.91 / 3.08 | 8.04 / 5.01 | 8.02 / 4.17 | 9.15 / 4.81 | 11.47 / 7.40 | x |
| **Muscle Cells** | x | 14.46 / 9.97 | 17.38 / 11.84 | x | x | 15.35 / 9.50 |
| **‍** |  |  |  |  |  |  |

**Table S3:** Probability for a surrogate CpG to be located in the same genomic segment as the clock CpG to be replaced. Definition of segments according to the chromHmm segmentation available at UCSC (https://genome.ucsc.edu) and shown in Table S5. Column “specific” refers to the exact segment, “general” if segments belonging to enhancer categories (resp. promoter categories or transcription categories) are pooled. In the case of general segments this probability is approximately stable up to the 10^th^ surrogate.

| **Level** | **specific** | **general** |
| --- | --- | --- |
| 1. | 0.352 | 0.636 |
| 2. | 0.370 | 0.582 |
| 3. | 0.297 | 0.594 |
| 4. | 0.297 | 0.612 |
| 5. | 0.261 | 0.618 |
| 6. | 0.224 | 0.558 |
| 7. | 0.242 | 0.533 |
| 8. | 0.164 | 0.570 |
| 9. | 0.188 | 0.521 |
| 10. | 0.285 | 0.594 |

**Table S4:** Distribution of the set of clock CpGs and their first surrogates in different chromHmm segments. Definition of segments according to the chromHmm segmentation available at UCSC (https://genome.ucsc.edu) and shown in Table S5.

| En-Pd | En-Pp | En-Sd | En-Sp | En-W | Hc-H | Hc-P | NS | Pr-A | Pr-B | Pr-F | Pr-W | Tr-I | Tr-P | Tr-S |
| --- | --- | --- | --- | --- | --- | --- | --- | --- | --- | --- | --- | --- | --- | --- |
| 12 | 3 | 2 | 5 | 1 | 0 | 16 | 13 | 13 | 33 | 9 | 14 | 2 | 25 | 17 |
| 6 | 2 | 2 | 6 | 1 | 1 | 19 | 14 | 11 | 38 | 6 | 11 | 4 | 21 | 23 |

**Table S5:** Relation between clock CpGs and their 1^st^ surrogates for the trained liver clock. Shown is the number of clock CpGs belonging to a chromHmm segment for the original clock (lines) and having a surrogate CpG in a specific chromHmm segment (columns). Definition of segments according to the chromHmm segmentation available at UCSC (https://genome.ucsc.edu).

|  | **Pr-A** | **Pr-W** | **Pr-B** | **Pr-F** | **En-Sd** | **En-Sp** | **En-W** | **En-Pd** | **En-Pp** | **Tr-S** | **Tr-P** | **Tr-I** | **Hc-P** | **Hc-H** | **NS** |
| --- | --- | --- | --- | --- | --- | --- | --- | --- | --- | --- | --- | --- | --- | --- | --- |
| **Pr-A** | 1 | 2 | 10 | 0 | 0 | 0 | 0 | 0 | 0 | 0 | 0 | 0 | 0 | 0 | 0 |
| **Pr-W** | 3 | 5 | 2 | 1 | 0 | 0 | 0 | 0 | 0 | 0 | 0 | 0 | 3 | 0 | 0 |
| **Pr-B** | 4 | 2 | 15 | 2 | 0 | 0 | 0 | 1 | 1 | 0 | 1 | 1 | 5 | 0 | 1 |
| **Pr-F** | 1 | 1 | 4 | 2 | 0 | 0 | 0 | 0 | 0 | 0 | 0 | 0 | 1 | 0 | 0 |
| **En-Sd** | 0 | 0 | 0 | 0 | 0 | 0 | 0 | 0 | 0 | 0 | 2 | 0 | 0 | 0 | 0 |
| **En-Sp** | 1 | 0 | 0 | 0 | 0 | 2 | 0 | 0 | 0 | 0 | 0 | 0 | 1 | 0 | 1 |
| **En-W** | 0 | 0 | 0 | 0 | 0 | 0 | 0 | 0 | 0 | 0 | 0 | 0 | 0 | 0 | 1 |
| **En-Pd** | 0 | 0 | 1 | 0 | 2 | 0 | 0 | 1 | 1 | 3 | 1 | 1 | 0 | 1 | 1 |
| **En-Pp** | 0 | 0 | 2 | 0 | 0 | 0 | 0 | 0 | 0 | 0 | 0 | 1 | 0 | 0 | 0 |
| **Tr-S** | 0 | 0 | 0 | 0 | 0 | 1 | 0 | 2 | 0 | 10 | 3 | 0 | 0 | 0 | 1 |
| **Tr-P** | 0 | 0 | 0 | 0 | 0 | 1 | 0 | 2 | 0 | 6 | 10 | 1 | 2 | 0 | 3 |
| **Tr-I** | 0 | 0 | 0 | 0 | 0 | 0 | 0 | 0 | 0 | 1 | 1 | 0 | 0 | 0 | 0 |
| **Hc-P** | 1 | 1 | 4 | 1 | 0 | 2 | 0 | 0 | 0 | 1 | 0 | 0 | 6 | 0 | 0 |
| **Hc-H** | 0 | 0 | 0 | 0 | 0 | 0 | 0 | 0 | 0 | 0 | 0 | 0 | 0 | 0 | 1 |
| **NS** | 0 | 0 | 0 | 0 | 0 | 0 | 1 | 0 | 0 | 2 | 3 | 0 | 1 | 0 | 6 |

| **Pr-A** | Promoter, Active |
| --- | --- |
| **Pr-W** | Promoter, Weak |
| **Pr-B** | Promoter, Bivalent |
| **Pr-F** | Promoter, Flanking Region |
| **En-Sd** | Enhancer, Strong TSS-distal |
| **En-Sp** | Enhancer, Strong TSS-proximal |
| **En-W** | Enhancer, Weak |
| **En-Pd** | Enhancer, Poised TSS-distal |
| **En-Pp** | Enhancer, Poised TSS-proximal |
| **Tr-S** | Transcription, Strong |
| **Tr-P** | Transcription, Permissive |
| **Tr-I** | Transcription, Initiation |
| **Hc-P** | Heterochromatin, Polycomb-associated |
| **Hc-H** | Heterochromatin, H3K9me3-associated |
| **Ns** | No significant signal |
| **‍** |  |

**Supplementary References**

Angermueller,C. et al. (2016) Parallel single-cell sequencing links transcriptional and epigenetic heterogeneity. Nature Methods, 13, 229–232.

Argelaguet,R. et al. (2019) Multi-omics profiling of mouse gastrulation at single-cell resolution. Nature, 576, 487-491.

Ficz,G., et al. (2013) FGF signaling inhibition in ESCs drives rapid genome-wide demethylation to the epigenetic ground state of pluripotency. Cell Stem Cell, 5, 351-359.

Hernando-Herraez,I. (2019) Ageing affects DNA methylation drift and transcriptional cell-to-cell variability in mouse muscle stem cells. Nature Communications, 10, 4361.

Kerepesi,C., et al. (2021) Epigenetic clocks reveal a rejuvenation event during embryogenesis followed by aging. Science Advances, 7, eabg6082.

Moqri,M. et al. (2024) PRC2-AgeIndex as a universal biomarker of aging and rejuvenation. Nature Communications. 15, 5956.
