# Supplementary material for: estiMAge: development of a DNA methylation clock to estimate the methylation age of single cells
Source: Bioinform Adv. 2025 Jan 16;5(1):vbaf005. doi: 10.1093/bioadv/vbaf005 (PMC11769677; doi:10.1093/bioadv/vbaf005)
Supplement: vbaf005_Supplementary_Data [file vbaf005_supplementary_data.zip › FigureS4.pdf]

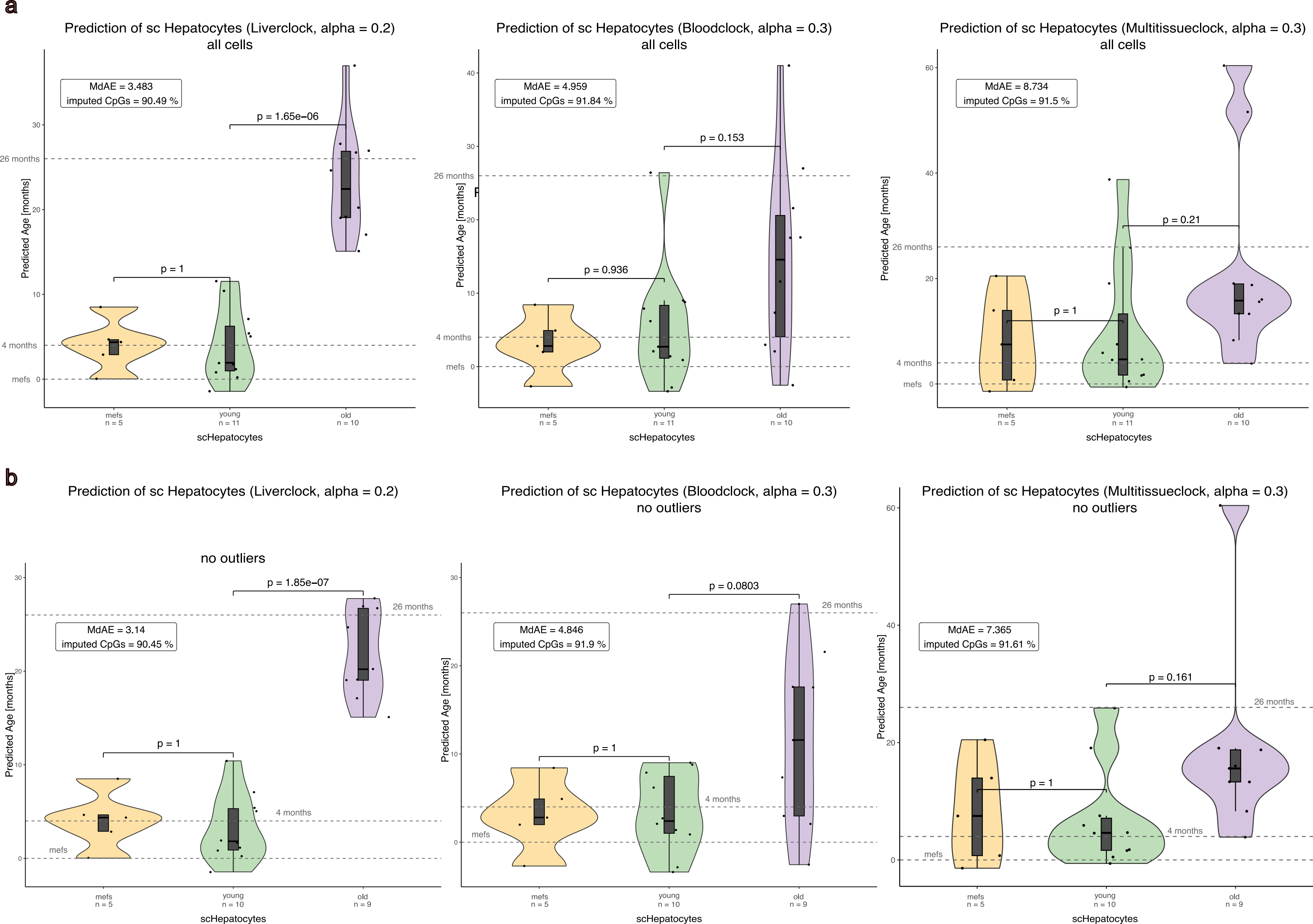
a
Prediction of sc Hepatocytes (Liverclock, alpha = 0.2)
all cells
MdAE = 3.483
imputed CpGs = 90.49 %
p = 1.65e−06
p = 1
Predicted Age [months]
26 months
4 months
mefs
mefs
n = 5
young
n = 11
old
n = 10
scHepatocytes
Prediction of sc Hepatocytes (Bloodclock, alpha = 0.3)
all cells
MdAE = 4.959
imputed CpGs = 91.84 %
p = 0.153
p = 0.936
Prediction of sc Hepatocytes (Multitissueclock, alpha = 0.3)
all cells
MdAE = 8.734
imputed CpGs = 91.5 %
p = 0.21
p = 1
b
Prediction of sc Hepatocytes (Liverclock, alpha = 0.2)
no outliers
MdAE = 3.14
imputed CpGs = 90.45 %
p = 1.85e−07
p = 1
young
n = 10
old
n = 9
Prediction of sc Hepatocytes (Bloodclock, alpha = 0.3)
no outliers
MdAE = 4.846
imputed CpGs = 91.9 %
p = 0.0803
p = 1
Prediction of sc Hepatocytes (Multitissueclock, alpha = 0.3)
no outliers
MdAE = 7.365
imputed CpGs = 91.61 %
p = 0.161
p = 1
