# Supplementary figures and images for: estiMAge: development of a DNA methylation clock to estimate the methylation age of single cells
Source: Bioinform Adv. 2025 Jan 16;5(1):vbaf005. doi: 10.1093/bioadv/vbaf005 (PMC11769677; doi:10.1093/bioadv/vbaf005)

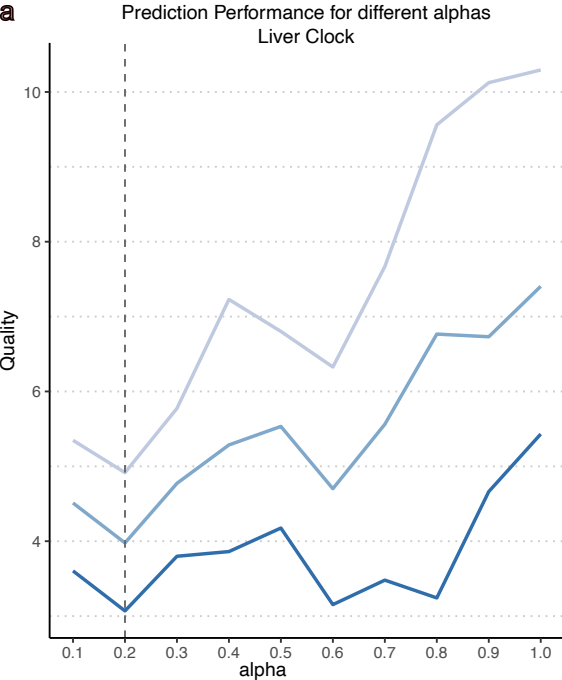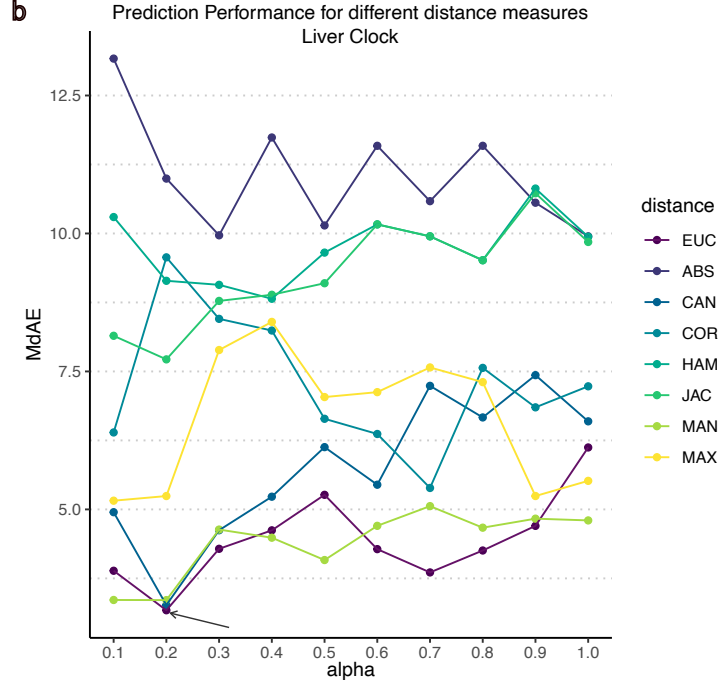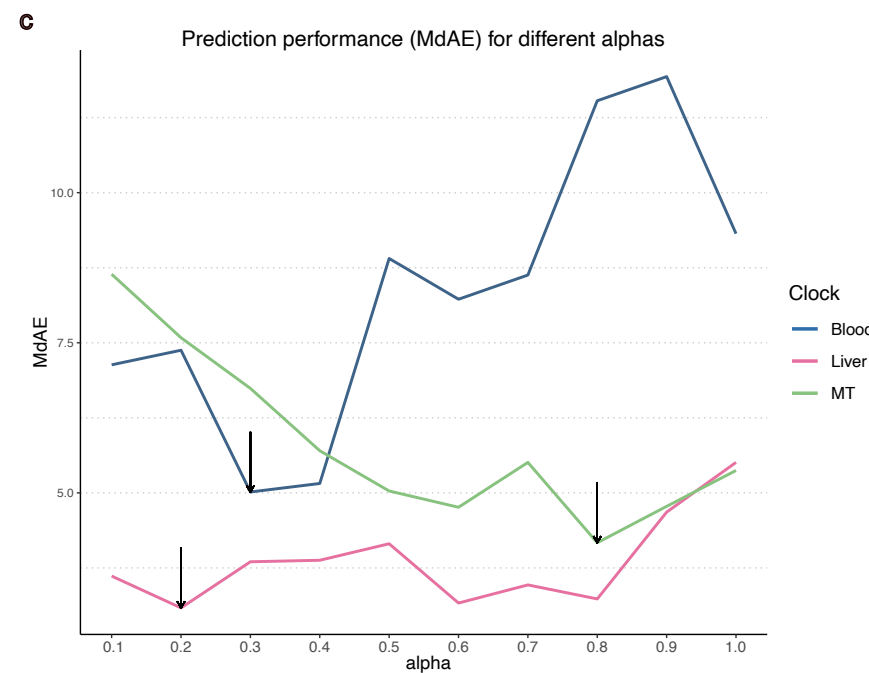

Supplement: vbaf005_Supplementary_Data [file vbaf005_supplementary_data.zip › FigureS2.pdf]

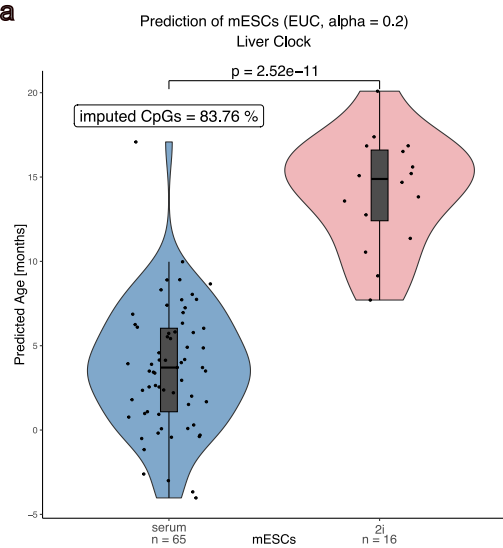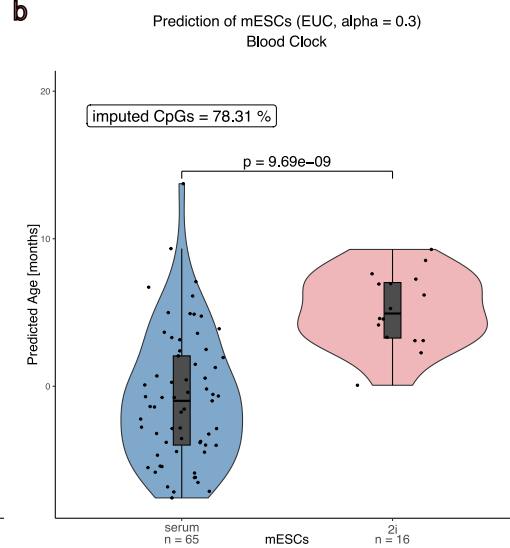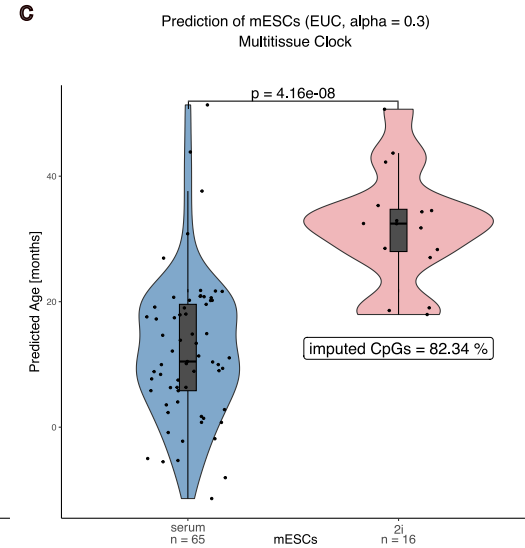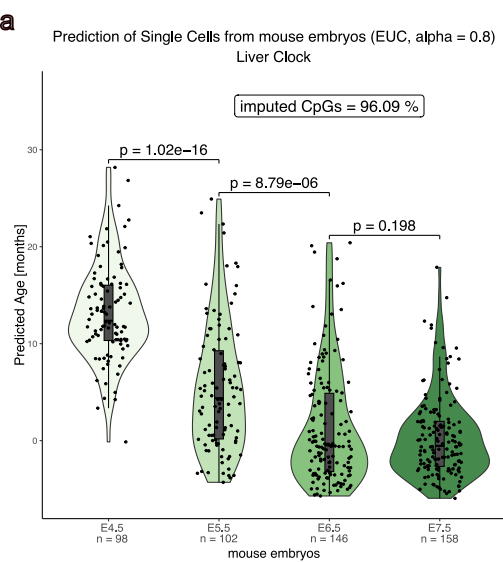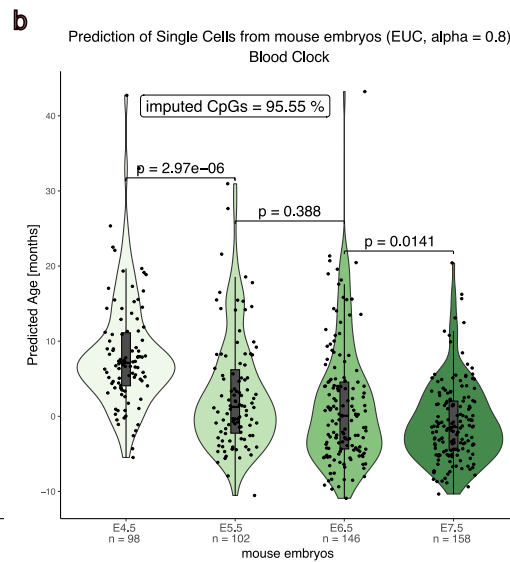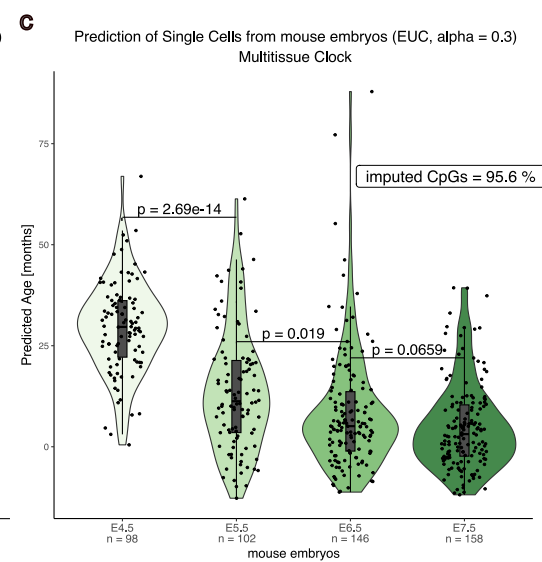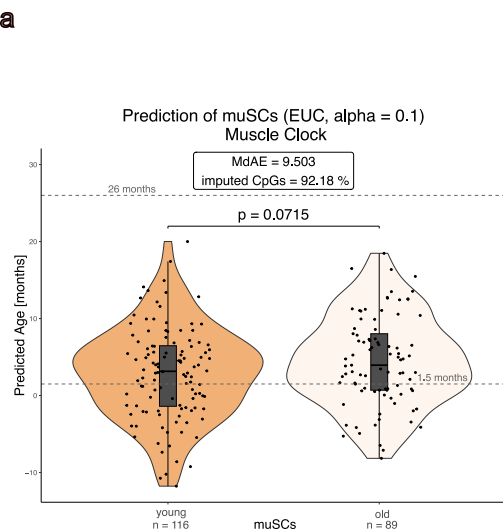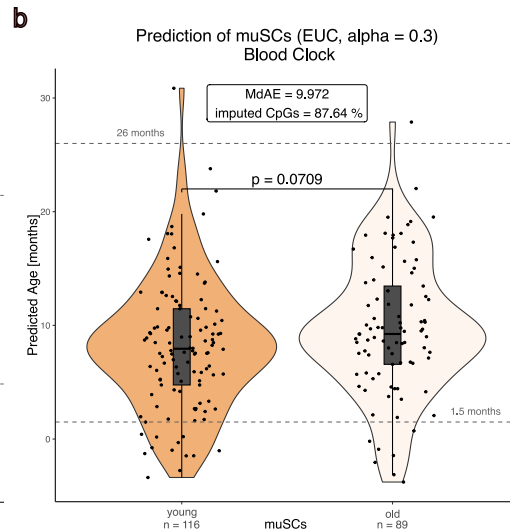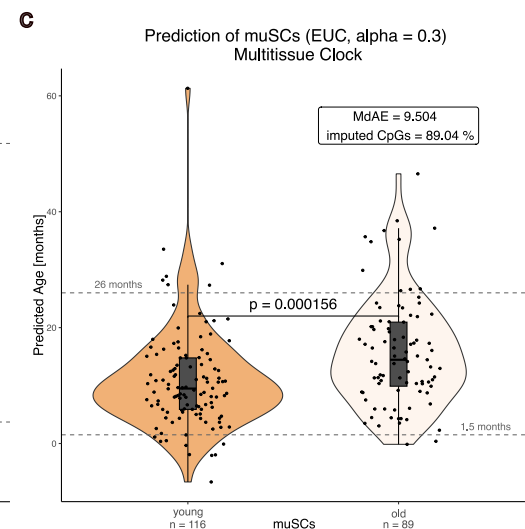

Supplement: vbaf005_Supplementary_Data [file vbaf005_supplementary_data.zip › FigureS1.pdf]

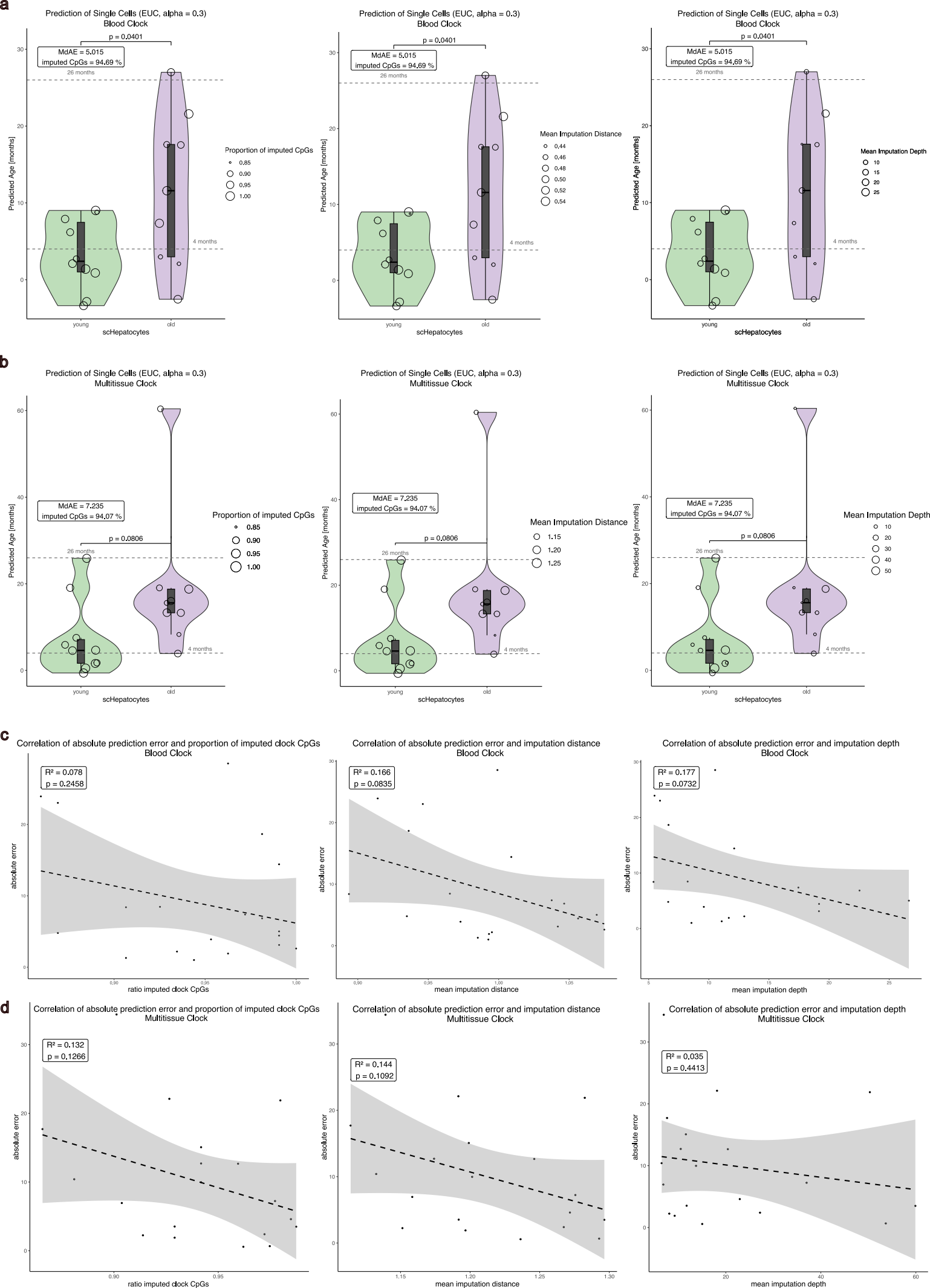

Supplement: vbaf005_Supplementary_Data [file vbaf005_supplementary_data.zip › FigureS3.pdf]
